# Field emission microscope for a single fullerene molecule

**DOI:** 10.1038/s41598-022-06670-1

**Published:** 2022-02-17

**Authors:** Hirofumi Yanagisawa, Markus Bohn, Florian Goschin, Ari P. Seitsonen, Matthias F. Kling

**Affiliations:** 1 JST, PRESTO, 4-1-8 Honcho, Kawaguchi, Saitama 332-0012 Japan; 2 Institute for Solid State Physics, The University of Tokyo, Chiba, 277-8581 Japan; 3 Physics Department, Ludwig-Maximilians-Universität Munich, 85748 Garching, Germany; 4 Max Planck Institute of Quantum Optics, 85748 Garching, Germany; 5 Département de Chimie, Ecole Normale Supérieure, 24 rue Lhomond, 75005 Paris, France

**Keywords:** Surfaces, interfaces and thin films, Condensed-matter physics

## Abstract

Applying strong direct current (DC) electric fields on the apex of a sharp metallic tip, electrons can be radially emitted from the apex to vacuum. Subsequently, they magnify the nanoscopic information on the apex, which serves as a field emission microscope (FEM). When depositing molecules on such a tip, peculiar electron emission patterns such as clover leaves appear. These phenomena were first observed seventy years ago. However, the source of these emission patterns has not yet been identified owing to the limited experimental information about molecular configurations on a tip. Here, we used fullerene molecules and characterized the molecule-covered tip by an FEM. In addition to the experiments, simulations were performed to obtain optimized molecular configurations on a tip. Both results indicate that the molecules, the source of the peculiar emission patterns, appear on a molecule layer formed on the tip under strong DC electric fields. Furthermore, the simulations revealed that these molecules are mostly isolated single molecules forming single-molecule-terminated protrusions. Upon the excellent agreements in both results, we concluded that each emission pattern originates from a single molecule. Our work should pave the way to revive old-fashioned electron microscopy as a powerful tool for investigating a single molecule.

## Introduction

**Figure 1:**
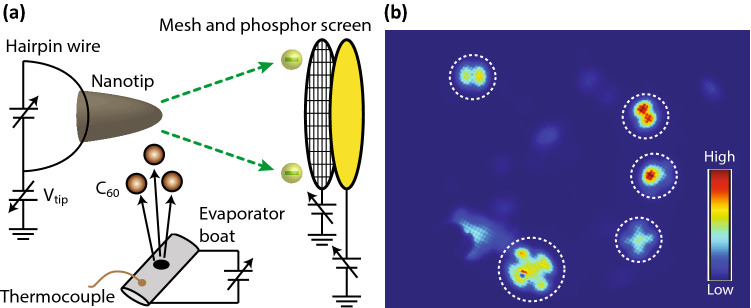
Experimental setup and typical FEM images of molecules. (**a**) Schematic diagram of our experimental setup of a field emission microscope. See the Method section for details. (**b**) Field emission patterns from single molecules. The patterns surrounded by dashed circles represent the molecule patterns.

A single molecule can be as small as a sub-nanometre in size, but even such a tiny molecule has functions such as single-molecule magnets^1–3^ or switches^4,5^. Investigating their physical properties in a single molecule requires a microscope with spatial resolution on the atomic scale. Hitherto it has often been thought that the most powerful microscope for a single molecule would be a scanning tunnelling microscope (STM). In an STM, electron current driven by DC voltages applied at the nanogap between a probe tip and a sample is measured. By scanning the tip over the sample, it enables one to visualize a single molecule with an atomic resolution^6^. An STM, however, requires an elaborate measurement setup with a good anti-vibration system, and hence its construction becomes expensive. In contrast, a field emission microscope (FEM), an old technique, is innately robust against vibration and can be realized by a relatively simple setup^7^. As shown in Fig. 1a, an FEM employs a tip with nanometre to sub-micrometre sharpness. By applying a high voltage between the tip and the metallic mesh, strong electric fields are concentrated at the tip apex, which induce electron emission from the solid to a vacuum. This is called field emission. The emitted electrons propagate radially from the tip apex. By mapping these electrons on a two-dimensional detector, one can project the nanoscopic information of a tip apex onto a macroscopic screen, which constitutes the microscope, namely the FEM.

By depositing molecules on the tip apex, molecules can be observed by the FEM. The spatial resolution of an FEM is typically one or two nanometres^7^. It is difficult to see the inner structure of a single molecule at the level of one nanometre. However, when a single molecule with a size of one nanometre appears on a tip apex, which is spatially separated from the other molecules, the magnification of the FEM can be increased by a factor of 20 and the spatial resolution becomes improved to around 3 Å^8^. Thus, it becomes possible to observe at least part of the inner structure of the molecules. Under this enhanced resolution, an FEM shows peculiar emission patterns from molecules such as a cross or a two-leaf pattern, as marked by the dashed circles in Fig. 1b. These peculiar patterns were already observed seventy years ago, but their interpretation was not clarified^7–13^. Because of this shortcoming, there are relatively few numbers of studies on molecules using FEM compared to STM. One of the reasons that prevent clarifying the interpretation is that it is not clear which configurations these molecules form on the tip apex and where the electrons come from. Previous studies with oxygen molecules revealed that these molecules form a layer on the tip apex, and on top of that layer, they form a nanoscale protrusion^10^. Around the protrusion, electric fields are further enhanced, and an electron emission is driven from the molecules at the top of the protrusions. However, it was not clear whether the molecules at the top are single or clustered^7–13^, unless one could undertake an exceptional study with an ultra-sharp tip^14^ or a single molecule emitter^15^. For instance, the authors in reference^10^ consider the protrusions to consist of a cluster of oxygen atoms, and each spot in the emission patterns representing a single atom. This interpretation, however, has not explained all the characteristics of the observed emission patterns.

Unlike an STM, an FEM shows only molecules with high electric fields at the top of protrusions. This limitation in the experimental information makes it difficult to reveal the detailed structures of the protrusions. In this study, together with our FEM experiments, we performed simulations to determine the optimized molecule configurations on a tip under strong DC electric fields, and thus, we aimed to learn about the sources of the emitted electrons. Here, we employed C_60_ molecules because of their highly isotropic properties. Based on its isotropic aspect, we assumed the molecule as a simple sphere in the simulations^16^ and could thus vastly reduce the computational cost. The experimental results indicated formations of protrusions on a buffer layer, as discussed in the previous work^10^. The simulations further revealed that each protrusion is primarily a single molecule, and the protrusion is the potential source of electrons.

## Results

### Experimental: summary of characteristic FEM images and our interpretations

**Figure 2:**
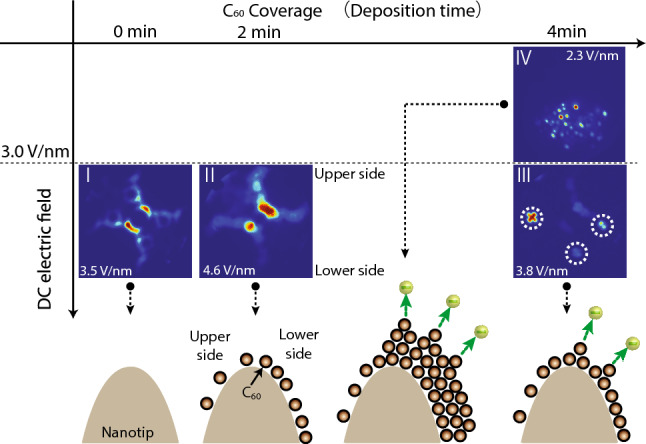
Summary of the deposition time and DC-electric-field dependences of FEM images together with their interpretations. Deposition time and DC-electric-field dependences of the FEM images of molecules. The temperature of the evaporator boat was 420 °C. The value of the DC electric field at the top of the tip apex, *F*_*DC*_ is denoted in each image. A schematic diagram of the interpretation of each image is also depicted. *F*_*DC*_ was determined using the following equation: $F_{DC} = \frac{{V_{tip} }}{kr},$ where *V*_*tip*_ is the voltage applied to the tip, *r* is the tip radius, and *k* is a parameter that varies depending on geometrical factors, such as tip shape, geometries of surrounding electrodes and polar angles of the sphere of the tip apex^7^. In this particular case, we determined *k*_*top*_, which is defined at the very top of the tip apex. The tip radius was separately measured once using a scanning electron microscope (SEM). In addition, before each experiment, we recorded threshold tip voltages at which the electron signals from the field emission could be seen on a phosphor screen. Here, we assumed that the *F*_*DC*_ for the threshold voltages is constant as long as the tip surface conditions, such as material and cleanliness, are the same. The threshold voltage is inversely proportional to the tip radius under this assumption. At each experiment, we determined the tip radius by multiplying the tip radius that was measured using the SEM by a proportion determined by the changes in threshold voltages. The parameter, k, was determined by fitting the theoretical FN curves provided in the references^18,19^ onto the experimentally obtained FN plots. The signals for the FN plots were taken from the (310) type facets of a tungsten tip, as indicated by the rectangles in the inset of Fig. 4e. There are three fitting parameters: temperature, work function and *k*. The previously obtained value of 4.45 eV was used for the work function^20^. The temperature was set to room temperature or 300 K. The $k$ parameter was determined by a curve fitting using the FN plots. Thus, the obtained *k* value for the (310) type facets was multiplied by a factor of 1.04 to obtain *k*_*top*_^7^. The uncertainty of *F*_*DC*_ determined in this method is 20 percent at most^7^.

We shall first examine the experimental results. After depositing molecules on a tip, different FEM images could be observed depending on the amount of deposited molecules and DC electric fields. These images are summarized in Fig. 2, together with the schematic diagrams of our interpretations. Deposition of molecules is done by resistively heating the evaporator boat, as shown in Fig. 1. In the experiments presented in this article, the temperature of the evaporator boat during the deposition was 420 °C and we gauged the amount of deposited molecules by a deposition time. A clean tungsten carbide sample was mainly used in our experiments (see the Method section for details on the experiments). The emission pattern from the tungsten carbide is shown in image I at the leftmost panel in Fig. 2. After depositing molecules for 2 min, the lower side of the tip became darker, as shown in image II. This is because of the formation of a molecular layer around the lower half of the apex, as schematically depicted below the FEM image. (We call this layer a buffer layer, which will be explained later.) Since the molecule source was installed below the tip, molecules were deposited mainly on the lower side of the tip. Note that we applied -500 V at the tip during the deposition. Under this condition, molecules are attracted towards the tip apex, and some molecules can be deposited even on the upper side^17^.

After depositing molecules for 4 min, peculiar emission patterns could be observed over the molecule layer, as shown in image III. It should be emphasized that the electron emission from the tungsten carbide surface could be slightly discerned from the upper half of the apex, and their signals got weaker in the lower half, indicating that there was a molecule layer mainly at the lower part of the apex. It should be also mentioned that the molecule layer was more firmly bonded to the surface than the molecules above the first layer, as we will discuss later. The molecules above the first layer are considered to be chemically pristine fullerenes (though, when the applied field is present, they may be partially charged and/or have a dipole moment) because previous studies have revealed that the electronic properties of molecules above the first layer are the same as that of the pristine C_60_, which was true for various metal substrates, including tungsten^21–23^. Hence, we call the first layer a buffer layer to distinguish it from the other layers. As depicted in the interpretation schematic for image III, those molecules on the buffer layer were well separated from each other, and thus they formed protrusions of single molecules. The peculiar emission patterns originated from the protrusions.

**Figure 3:**
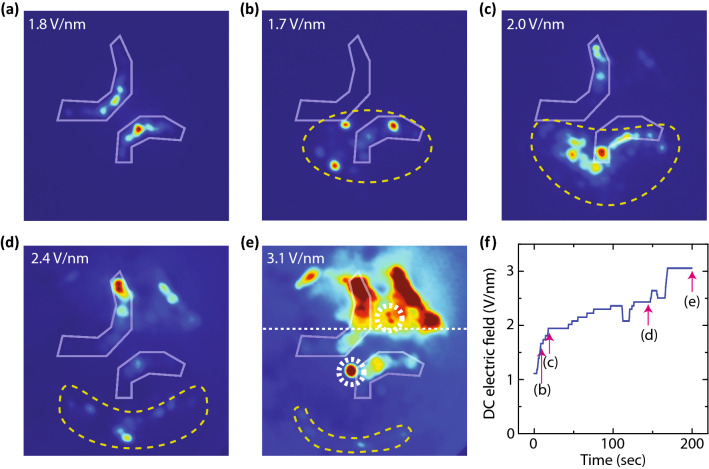
Evaporation of weakly-bound molecules by DC electric fields. (**a**) FEM image of clean tungsten carbide. (**b**)–(**e**) FEM images with increasing DC electric fields after the deposition of molecules. The electric fields are shown in the images. The electron signals from the weakly-bound molecules are surrounded by dashed lines. The emission areas of the tungsten carbide are highlighted by white lines. In (**e**), the peculiar molecule emission patterns are highlighted by dashed circles. (**f**) Applied DC electric fields and their corresponding timestamps in the experiments.

Under the same deposition time but with lower DC electric fields, the FEM image became different, as shown in image IV in Fig. 2. In this image, a flock of random and tiny spots on the lower half of the image can be observed. We believe that several molecule layers were formed on the tip, and electrons were emitted from some protrusions that appeared on the topmost layer, as depicted in the figure. Those molecules above the first layer were weakly bound to the surface, and they were evaporated under strong DC electric fields. This transition from the weak DC electric fields to the strong DC electric fields can be observed by a series of FEM images, as shown in Fig. 3.

### Experimental: evaporation of molecules by DC electric fields

Figure 3a is an FEM image of clean tungsten carbide. For illustrative purposes, the emission areas of the tungsten carbide have been highlighted by white lines, and the same lines are indicated in Fig. 3b–e. After depositing molecules for 4 min, we saw a flock of tiny spots under a weak DC electric field, as shown in Fig. 3b. These spots are surrounded by dashed lines. With increasing electric fields, the flock of spots gradually disappeared from the top of the tip apex, and the dashed-line area gradually moved downwards, as shown in Fig. 3c–e. As the flock of spots disappeared, the signals from the substrate became visible. The applied electric fields and the corresponding timestamp of related experiments are shown in Fig. 3f. At the highest fields in Fig. 3e, we could observe the peculiar emission patterns such as a large round spot and a two-leaf pattern surrounded by dashed circles. Additionally, the electron signals become weaker below the dashed line, which indicates that a buffer layer remains mainly below the line. This is the same situation as image III in Fig. 2. It should be mentioned that the dashed line in Fig. 3e does not indicate the clear boundary of the buffer layer but indicates that the density of molecules in the buffer layer becomes sparse above the line. The buffer layer molecules should exist even on upper side of the tip as explained in image II of Fig. 2. We believe a molecule that is the source of the two-leaf pattern in Fig. 3e is situated on a buffer layer, though the pattern appeared above the dashed line. The reason for this is that we have rarely seen those peculiar patterns at the low coverages where the formation of the buffer layer cannot be observed.

These results in Fig. 3 indicate that the flocks of spots were signals from the molecules on the buffer layers, and they were weakly bound to the surface. These weakly-bound molecules would be forming more than a monolayer on the buffer layer as the emission patterns were rather different from the ones on the buffer layer. Note that these molecules on the buffer layer consistently evaporated from the top of the apex, because the DC electric field was the strongest at the top of the tip apex and got weaker toward the shank side.

### Experimental: evaporation of molecules by heating

**Figure 4:**
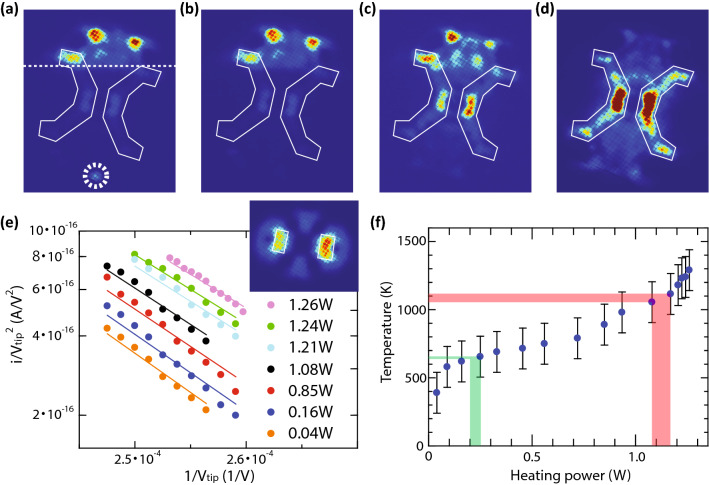
Evaporation of strongly-bound molecules by heating. (**a**)–(**d**) A series of FEM images with increasing temperature of the tip. The temperatures are sequentially increasing. The image in (**a**) was taken at room temperature. The emission areas of the tungsten carbide are highlighted by white lines. In (**a**), a peculiar molecule emission pattern is marked by a dashed circle. (**e**) Heating power dependence of FN plots. The inset shows an FEM image of a clean tungsten surface. The signals for the FN plots are taken from (310) type facets as indicated by the rectangles in the inset. The vertical axis in figure (**e**) is signal *i* over the $V_{tip}^{ 2}$ on a logarithmic scale. The signal *i* used in figure (**e**) is the total yield of electron emission from the right rectangle in the inset. The horizontal axis is $1/V_{tip}^{ } .$ The temperature of the tip was extracted by fitting theoretical FN curves given in the references^18,19^ onto experimentally obtained FN plots at each heating power. There are three fitting parameters: a temperature, a work function and a voltage conversion factor $C_{L}$, where the applied voltage $V_{tip}^{ }$ can be converted to a local electric field $F_{L}$ via $F_{L} = C_{L} V_{tip}$. The unit of $F_{L}$ is V/nm. As described in the caption in Fig. 2, $C_{L}$ is expressed by $C_{L} = \frac{1}{kr}$. In this series of experiments, the tip radius r was 165 nm. The parameter *k* was determined by a curve fitting using the FN plots at room temperature. The resulting value of *k* was 6.91 and hence the value of $C_{L}$ was $8.77 \times 10^{ - 4}$. For the work function, the previously obtained value of 4.45 eV was used^20^. Then the temperature was determined for the FN plots at each elevated heating power via the curve fittings. The resulting FN curves are shown as solid lines in figure (**e**) by using the same colour code as those of the corresponding FN plots. (**f**) Plots of temperature versus heating power. The temperatures were extracted from the FN plots from both rectangle areas in (**e**) and were calculated by taking an average of the two cases. The width of the colour bands indicate the ranges of heating powers where the molecule patterns (green bands) and the buffer layer (red bands) disappeared.

The buffer layer could not be removed by the DC electric fields though we tried up to around 4.5 V/nm as in image II of Fig. 2. However, it could be removed by heating the tip. The variations of FEM images under increased heating power are shown in Fig. 4b–d. The emission areas of the tungsten carbide are surrounded by white lines for a better visibility. The deposition time was 4 min. In Fig. 4a, a molecule emission pattern marked by a dashed circle can be observed at room temperature, and the buffer layer can also be observed as the darker area spreads below the dashed line. The surface condition in this case was the same as that in image III of Fig. 2. With increasing heating power, the marked molecule pattern disappeared, as shown in Fig. 4b. Further increasing the heating power caused the substrate signal to increase, and the difference between the upper and lower sides also disappeared, as shown in Fig. 4c and d, which indicates the evaporation of the buffer layer from the apex. We also have estimated the temperature when the molecule patterns and the buffer layer disappeared. The temperature at the tip apex was extracted from Fowler–Nordheim (FN) plots in Fig. 4e. The signals of the FN plots were taken from the emission from (310) type facets of a clean tungsten surface as indicated by rectangles in the inset of Fig. 4e. The FN plots change with increasing heating power because of their dependency on temperature^18,19^. From the power-dependent FN plots, we successfully extracted the sample temperatures and plotted them in Fig. 4f. In addition, we recorded the heating power when the molecule patterns and the buffer layer disappeared. We repeated the same experiments a couple of times. The ranges of the resulting powers are indicated by bands in Fig. 4f, and we determined their temperature from the plots. The results show that the molecular patterns disappear at around 640–660 K (green bands), and the buffer layer disappears at around 1050–1120 K (red bands). The previous work showed that the evaporation temperature of the weakly-bound layers was around 570 K^24^. So the molecules, the sources of the peculiar emission patterns, are more or less as weakly bound to the buffer layer as the weakly-bound layers in image IV of Fig. 2. In contrast, the evaporation temperature of the buffer layer is roughly two times higher than that of the weakly-bound layers.

### Simulations: quick overview of characteristics of dipoles

**Figure 5:**
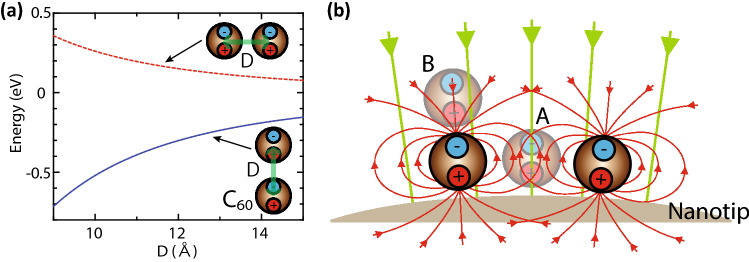
Characteristics of dipole interactions and fields. (**a**) Variations of dipole–dipole interaction energy under an electric field of 4 V/nm with changing *D* for horizontally- and vertically-aligned dimers, where *D* is the distance between two molecules. (**b**) Schematic diagram of the lines of the field forces of the induced dipole fields (red lines) and the DC electric fields around a tip (green lines).

In order to have a clearer vision of the molecule configurations on a tip, we have simulated optimized molecule configurations on a tip under strong DC electric fields. The details of these interactions and parameters used in the simulations are given in the Method section. In the simulations, we ignored the atomic structures of C_60_ and regarded it as a simple sphere. Under DC electric fields, C_60_ will be polarized and generate a dipole. The interactions associated with the dipoles govern the molecule configurations under strong electric fields. We shall first examine a couple of characteristics of dipoles. First, as shown in Fig. 5a, if two dipoles are horizontally aligned, they will undergo repulsive forces from each other. In contrast, if they are aligned vertically, they will become attracted to each other. These aspects indicate that molecules at the same height will tend to stay apart from each other, and they prefer to be aligned vertically if dipoles are induced in them. Another important factor is the total energy of a dipole^25,26^. This energy draws a dipole, or a molecule, to a position with higher electric fields^27^. We shall see the characteristics of the electric field distributions. Around the tip, strong DC electric fields radially spread from the apex along their lines of field force, as illustrated by the green lines in Fig. 5b. The induced dipoles will modify the electric fields around the apex. For instance, we shall assume four molecules on a tip apex as drawn in Fig. 5b. The electric fields at position A will be reduced by the dipole fields in the two molecules beside it because the signs of the dipole fields are opposite to those of the DC electric fields. In contrast, the electric fields are enhanced at position B because the sign of the dipole fields is the same as that of the DC fields. This means that the single molecule on a buffer layer has stronger electric fields than the molecules in the layer beneath. Because of the characteristics mentioned above, molecules would tend to form a protrusion under strong DC electric fields, and the protrusion becomes the potential source of electrons as it will have high electric fields. It should be noted that the normal explanation of field enhancement above a surface protrusion involves charging of the protrusion^28^. In this simulation, since the charging effect was ignored, the enhanced electric fields at a protrusion are supposed to be underestimated. To our minds, further electric-field enhancement at a protrusion due to the charging effect would further stabilize the protrusion by enhancing a dipole moment at the protrusion molecule. We believe that the further stabilization would not significantly affect our conclusion of the formation of a single molecule protrusion below inferred from our simple dipole model. In order to achieve more quantitatively accurate simulations, the development of simulation models based on density functional theory is required.

### Simulations: optimized molecule structures under DC electric fields

**Figure 6:**
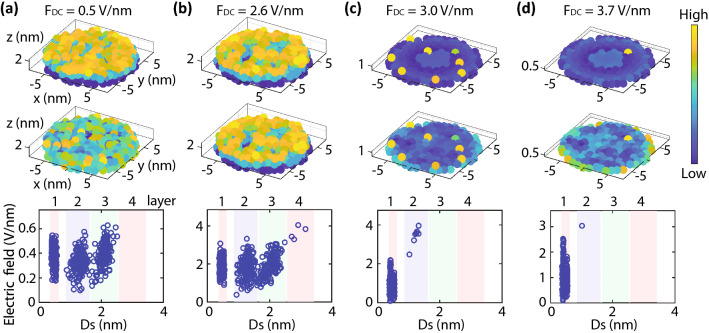
DC-electric-field dependence of optimized molecule configurations. (**a**)–(**d**) Optimized molecule configurations on a tip with increasing DC electric fields, *F*_*DC*_. *F*_*DC*_ is the value of the electric fields at the very top of the tip apex. The values of *F*_*DC*_ are denoted at the top of the figures. The colour in the upper panels represents the height of molecules with respect to the tip surface, *Ds*. The colour in the middle panels represents the strength of the electric fields. The lower panel shows plots of electric fields versus *Ds*. The range of each layer is highlighted by the different colour bands for convenience.

In the simulations, we have randomly deposited 500 fullerenes on a tip apex with its curvature at a radius of 160 nm and formed three layers. Here, we assumed the atomic structures of tungsten for the model tip, and the tip apex is oriented towards a [001] direction. An optimized structure under weak DC electric fields, *F*_*DC*_ of 0.5 V/nm, is shown in Fig. 6a. The colour of the upper panel represents the height of molecular centres (sphere centres) from the surface of the sphere of the tip apex, *Ds*. The colour of the middle panel represents the strength of the electric fields at the centres of the molecular spheres. In the lower panel, these electric fields are plotted against *Ds*. One can quickly confirm that the molecules are piled up to the third layer in the lower panel. We have simulated optimized structures with increasing DC electric fields step by step. At an *F*_*DC*_ of 2.6 V/nm, in Fig. 6b, four molecules jumped up to the fourth layer and formed protrusions. As can be seen in the middle and lower panels, the protrusions had stronger electric fields than other molecules beneath. Those molecules with high electric fields would emit electrons, which would be the same situation as image IV of Fig. 2. At an *F*_*DC*_ of 3 V/nm, in Fig. 6c, most of the molecules above the first layer evaporated due to the wind force, which is a force caused by the field emission from a molecule. This is the evaporation of the weakly-bound layers, which was observed in the experiments. Note that the weakly-bound layers evaporated around 3.0 V/nm in the experiments, as shown in Fig. 3. The agreement is remarkable, though the wind force is a very simple model^29,30^. For a more accurate descriptions of the physics, one has to simulate using a density functional theory^31^.

**Figure 7:**
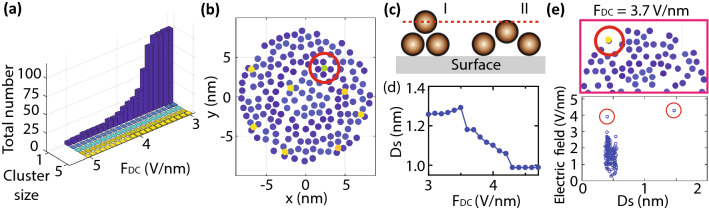
Molecule configurations at the high electric fields. (**a**) DC-electric-field dependence of a histogram of cluster size. The size of a cluster was determined by counting the number of molecules that were closely connected with each other. If two molecules are located within 11 Å, we defined them as connected. (**b**) Front view of the upper panel in Fig. 6c. The molecule marked by a red circle is the one remaining in Fig. 6d. (**c**) A schematic diagram of two kinds of molecule protrusions with different configurations. (**d**) The DC-electric-field dependence of average *Ds* at the second layer. (**e**) Another example of a molecule protrusion at high electric fields. Optimized molecule configurations on a tip (upper panel) and the plots of electric fields versus *Ds* (lower panel). The colour of the upper panels represents *Ds*.

After the evaporation of the weakly-bound layer, some molecules remained on the first layer, as shown in Fig. 6c. Those molecules on the first layer were separated from each other and formed single-molecule-terminated protrusions. At even higher electric fields, in Fig. 6d, most of the molecules evaporated from the second layer, and only one molecule remained. Those protrusions in Fig. 6c and d have higher electric fields compared to others in the first layer, and hence they would be the potential sources of electron emission. We repeated the series of simulations 15 times and confirmed that most of the remaining molecules on the first layer were isolated from each other, forming single-molecule-terminated protrusions when the field strength was stronger than 3 V/nm. It should be mentioned that there occasionally appeared clusters of some molecules. As shown in the histogram of cluster size with varying DC electric fields in Fig. 7a, one cluster with five molecules and one with a dimer appeared once in 15 trials. (See the figure caption for how to count the number of molecules in a cluster.) In contrast, isolated single molecules appeared around 100 times at 3 V/nm. Because the majority of the emission patterns in FEM images under strong electric fields are peculiar molecule patterns, we concluded that each peculiar pattern originated from a single molecule.

## Discussion

We would like to point out configurational differences around single-molecule protrusions. As can be seen in Fig. 7a, some single molecules of protrusions evaporated around 3.5 V/nm and some remained at up to 5 V/nm. The difference in the electric fields for evaporation originates from the molecule configurations. For instance, one of the molecules in Fig. 6c, which remained even at the higher electric fields in Fig. 6d, is marked by a red circle and shown in its front view in Fig. 7b. The difference between the marked molecule and the others is the alignment of molecules in the first layer, as schematically drawn in Fig. 7c. Those molecules which evaporated around 3.5 V/nm are among case I, where the molecules in the first layer are closely located to each other. Case I includes configurations such as a molecule sitting on a hollow site, bridge site and on-top site. In contrast, the molecules that remained at the higher electric fields are in case II, where the molecules in the first layer had some spaces. Note that this configuration resembles the previously observed C_60_ island with apexes^32^. These two cases are gauged by *Ds*, since *Ds* in case I is higher than that of case II. This tendency can be clearly seen in Fig. 7d. The average height of the topmost molecule is decreasing with increasing *F*_*DC*_. Thus, the value of *F*_*DC*_ needed to induce molecule evaporation depends on the height of the topmost molecule above the surface, with low height corresponding to high *F*_*DC*_, and vice-versa. The molecules in case I evaporated first because (at all applied voltages) the local electric fields at the topmost molecules were higher in case I than in case II, due to the molecule configurations. When the local electric field is sufficiently high, then the local field emission current is sufficiently high, the local wind force is sufficiently high, and evaporation occurs.

It should be emphasized that there are exceptional cases where protrusion of the case I group remains even at higher electric fields. As marked by a red circle in the upper panel of Fig. 7e, such a protrusion was situated at the edge of the molecule layer. In this protrusion, a molecule was situated at an on-top site, forming a vertically aligned dimer. As shown in Fig. 5b, if a molecule is surrounded by other molecules at the same level, the electric fields at the centre molecule will be reduced due to the dipole fields from surrounding molecules. This effect will be mitigated at the edge of a layer. This aspect can be clearly seen in the lower panel of Fig. 7e, where it shows plots of electric fields versus *Ds*. The two molecules of the protrusion are marked by red circles. The electric field of the marked molecule in the first layer was higher than the others in the first layer. This high electric field created a stronger attraction force in dipole–dipole interaction. As a result, it could remain even in higher electric fields.

In the end, we would like to comment on the emission patterns. As discussed above, the protrusions had different configurations. All such protrusions are considered to be the potential source of electrons. Here, a question arises: Are the molecule emission patterns influenced by the difference in the molecule configuration in the first layer? We believe that the answer is Yes. These emitted electrons were originally coming from the substrate metal, and they passed through molecules before emission. The emission patterns would reflect molecular orbitals that electrons pass through. Therefore, for instance, if a topmost molecule was at an on-top site, the emission pattern from that topmost molecule would reflect the molecular orbitals of two molecules in the first and the second layers. In contrast, the emission from the topmost molecule in case II in Fig. 7c would be less affected by the molecule in the first layer because there was no molecule just beneath the topmost molecule. In this case, the molecular orbitals in a single fullerene could be investigated.

In this article, we have characterized the apex of a molecule-covered tip at different amounts of molecules and DC electric fields from an FEM. At the high amount of molecule deposition and under DC electric fields below 3 V/nm, weakly-bound molecules covered the buffer layer. At the same deposition amount and under stronger DC electric fields, the weakly-bound molecules were evaporated, and we confirmed that the molecules, which were the source of the peculiar emission patterns, appeared on a molecule buffer layer. The simulations on optimized molecule configurations on a tip reproduced the same phenomena. Also, they revealed that molecules on the buffer layers were mostly spatially separated from each other, forming single-molecule-terminated protrusions under strong DC electric fields. The simulations further showed the possible configurations of protrusions. These results are helpful to construct a theoretical model to understand the mechanism of electron emission from a single molecule on a tip. FEMs have been applied for investigating molecules many times over the past seven decades. Our study finally showed that an FEM is a simple and useful tool to extract electron signals from a single molecule. One does not need to create a special condition like an ultrasharp tip to realize an electron emission from a single molecule. This conclusion can be applied only to fullerene molecules in this study, but the same conclusion could be drawn for other molecules as well because the appearance of the emission patterns is the same among other molecules. Our results would be useful in reveal the interpretation of peculiar emission patterns and could pave the way to rekindling the FEM as a powerful tool to study a single molecule.

## Methods

### Experimental

Field emission experiments were performed in an ultra-high vacuum chamber. The base pressure was $1 \times 10^{ - 10}$ mbar. A tip and a phosphor screen were installed in the chamber to perform FEM experiments, as shown in Fig. 1. A metallic mesh was installed in front of the phosphor screen. In order to induce field emission, the mesh and phosphor screen were grounded, and the tip was negatively biased except in the experiments depicted in Fig. 4. In the series of experiments shown in Fig. 4, the tip was heated by heating a hairpin wire on which the tip was placed, and the phosphor screen and mesh were positively biased to generate field emission from the tip. A chevron-type microchannel plate was installed between the mesh and the phosphor screen for the low DC-electric-field experiments depicted in image IV in Fig. 2 and the series of experiments shown in Fig. 3. As shown in Fig. 1, fullerene molecules were enclosed in an evaporator boat with a hole, which was installed beneath the tip. Molecules were evaporated from the hole by resistively heating the boat. A thermocouple was used to monitor the temperature of the evaporator boat. A voltage of − 500 V was applied to the tip during the deposition of molecules. Note that we have also attempted the deposition at a positive voltage. With positive voltages, we observed fewer molecular patterns in an FEM image. This tendency was pronounced with increasing voltages. For instance, after deposition at a positive voltage of 1500 V, even the formation of a buffer layer could not be observed, while we could observe many molecular patterns after deposition at − 1500 V. According to the theory of a gas supply process in the field ion microscope (FIM), the polarized gas molecules are attracted to the shank region near the apex and then move towards the tip apex by some sort of hopping motion^27^. In our case with positive voltages, we believe that molecules could be deposited on the shank region, but they could not move towards the tip apex because of the weak electric fields around the tip apex compared to those in FIM.

In the experiments, we used a tungsten tip with a crystallised apex oriented towards the [011] direction. The surface condition of the apex can be assessed using FEM^33^. A clean tungsten tip surface can be obtained by heating the sample. The inset of Fig. 3e shows a typical FEM image of the clean tungsten tip. In the experiments, we frequently repeated molecule deposition on the tip and removed the molecules by heating the tip. The process converted the tungsten surface to a tungsten carbide surface, resulting in a typical FEM image, as shown in image I in Fig. 2. The tungsten carbide could be removed from the surface by annealing the tip under an oxygen atmosphere. However, because there were no remarkable differences in the appearance of molecular patterns between both surfaces, we mainly performed experiments with a tungsten carbide tip. The radius of curvature of the tips used in this study ranged from 150 to 250 nm.

### Theoretical

Optimized molecular structures on a tip under strong DC electric fields are simulated by minimizing the energy of a molecular system. Here we included five different kinds of energies, along with a force. They are: (1) interaction energy between C_60_ and C_60_; (2) interaction energy between C_60_ and a tip surface; (3) dipole–dipole interaction energy among molecules; (4) interaction energy between image dipoles in the metal and dipoles in the molecules; (5) total energy of a dipole; and (6) a wind force. The third to sixth terms appear under DC electric fields. The last term is a force exerted on a molecule when electron currents are induced at the molecule due to field emission^29,30^.

### First and second energy terms

**Figure 8:**
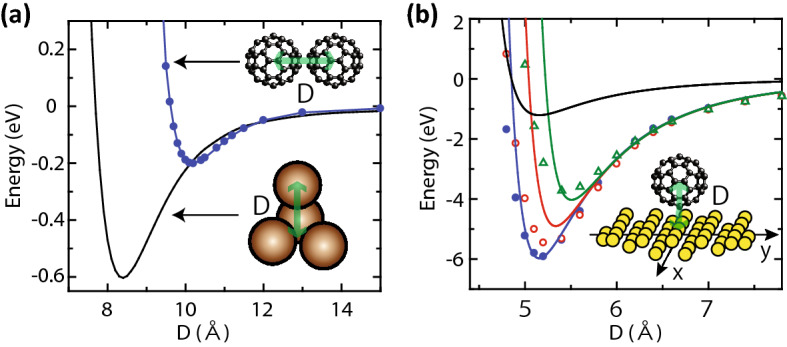
Variations of interaction energies. (**a**) The variations of C_60_–C_60_ interaction energy of a dimer and a tetramer with respect to their distance *D*. (**b**) Variations of the interaction energy between the C_60_ and tungsten surface. See the Method section for details.

The first two terms are calculated based on a density-functional theory using Quantum Espresso^34,35^. In the simulation, the non-local van der Waals functional (vdW-DF2-B86r) was applied^36^. In Fig. 8a, the resulting interaction energy of C_60_—C_60_ is plotted as a function of their distance *D*. Each C_60_ has its dimer face towards the other as schematically drawn in Fig. 8a. In the optimization simulation, we determined the interaction energy of C_60_—C_60_ by interpolating the resulting plots in Fig. 8a. For the interaction energy of the C_60_—tungsten surface, we first simulated energy variations with different positions of C_60_ with respect to the substrate atomic configurations to introduce the surface atomic corrugations into the simulations. The actual geometry of tungsten atoms is shown in the inset of Fig. 8b. The tungsten surface is orienting toward [001]. In Fig. 8b, the resulting interaction energies of the C_60_—tungsten surface are plotted as a function of *D* for three different positions. The open triangles are the results when the C_60_ is on an on-top site at (*x*, *y*) = (0, 0). The schematic diagram in the figure shows the atomic configurations for the on-top site simulation. The open circles are the results when the C_60_ is at (*x*, *y*) = (0, *L*/2), where *L* is the length of the unit vector. C_60_ is at a bridge site in this case. The solid circles are the results when the C_60_ is at (*x*, *y*) = (*L*/2, *L*/2). C_60_ is at a hollow site in this case. Using these results, we then, determined a Lennard-Jones (LJ)-type potential between a C_60_ sphere and a tungsten atom. The parameters of the LJ-type potentials were tweaked to have similar energy variations as those obtained by the DFT simulations. The resulting curves are shown by the same colour code as those of the corresponding plots. The obtained LJ-type potential was as follows: $V_{LJ1} = 4\epsilon_{0} \left( {\left( {\frac{5.4}{x}} \right)^{40} - \left( {\frac{5.4}{x}} \right)^{9} } \right)$, where *x* is the distance between a C_60_ sphere and a tungsten atom. In the experiments, the tungsten carbide surface was mainly used instead of tungsten. So we modified the obtained potential formula to mimic the experimental situation. From the heating evaporation experiments in Fig. 4, we have learned that the evaporation temperature of the buffer layer is approximately two times higher than that of weakly-bound molecules. The interaction energy for the weakly-bound molecules is roughly estimated by the case of a tetramer, as shown in Fig. 8a. The solid black lines indicate the interaction energy of the tetramer as a function of *D*. Then, the blue energy curve obtained for the hollow site in Fig. 8b was scaled down in such a way that the potential depth of the curve becomes double that of the tetramer curve, which is shown by the black curve in Fig. 8b. The scaling factor to the original blue curve was 0.2. Hence, we used the following formula for the interaction energy between C_60_ and a substrate atom: $V_{LJ2} = 0.8\epsilon_{0} \left( {\left( {\frac{5.4}{x}} \right)^{40} - \left( {\frac{5.4}{x}} \right)^{9} } \right)$. The strength of the interaction energy between C_60_ and a substrate atom was not important to our conclusion as long as it is significantly stronger than those of weakly-bound molecules so that all molecules do not evaporate at the low DC electric fields. Note that here we assumed the atomic structures of the tungsten (100) surface for the tungsten carbide surface and assumed all the substrate atoms were the same kind because the detailed structure of the tungsten carbide surface is not known. This factor would not very important as long as the atomic corrugation was introduced. Without the atomic corrugation of a substrate, molecules in the buffer layer can be slid in directions parallel to the substrate surface, no matter how strong the interactions are. The introduction of the surface atomic corrugation was to avoid such an unrealistic behaviour.

### Third, fourth and fifth energy terms

The dipole–dipole interaction energy, $V_{dip - dip}$ for the third term is calculated using the following formula^37^: $V_{dip - dip} = \frac{1}{{4\pi \epsilon_{0} r_{ij}^{3} }}\left[ {\vec{\mu }_{i} \cdot \vec{\mu }_{j} - 3\frac{{\vec{\mu }_{i} \cdot \left( {\vec{r}_{i} - \vec{r}_{j} } \right)\left( {\vec{r}_{i} - \vec{r}_{j} } \right) \cdot \vec{\mu }_{j} }}{{r_{ij}^{2} }}} \right]$, where $\epsilon_{0}$ is the dielectric constant, $\vec{r}_{i}$ and $\vec{r}_{j}$ are the position vectors for the two dipoles, $r_{ij}$ is given by $r_{ij} = \left| {\vec{r}_{i} - \vec{r}_{j} } \right|$ and $\vec{\mu }_{i}$ is given by $\vec{\mu }_{i} = \alpha \vec{F}_{i}$. $\vec{F}_{i}$ is the electric field at the position $\vec{r}_{i}$, and $\alpha$ is a molecular polarizability. We employed the molecular polarizability of 106.3 meV/(V/nm)^2^ (i.e. ${\upalpha }/4{\uppi }\epsilon_{0}$ = 153.1 Å^3^), which is calculated in the reference^38^. Note that meV/(V/nm)^2^ is a polarizability unit compatible with the modern $\epsilon_{0}$-based equation system used to define SI units, whereas Å^3^ is a unit of Gaussian polarizability, as used in the Gaussian-cgs equation system^39^. $V_{dip - dip}$ was calculated among the dipoles in molecules around the tip apex.

For the fourth term, namely the interaction energy between the image dipoles in the metal and dipoles in the molecules, we simulated the positions and amounts of classical image dipole charges for a metallic sphere. The image of an electric point dipole consists of not only an image dipole but also an image charge^40^. Suppose that there is a dipole with a dipole moment of $\vec{\mu }_{i}$ at $\vec{r}_{i}$ on the tip surface, the image dipole and charge in the metallic sphere are located at $\vec{p}_{im} = \left( {\frac{{R_{surf}^{2} }}{{r_{i}^{2} }}} \right)\vec{r}_{i}$, where $r_{i}$ is $\left| {\overrightarrow {{r_{i} }} } \right|$ and $R_{surf}$ is the distance between the tangential image plane and the center of the sphere. $R_{surf}$ is given by $R_{surf}^{^{\prime}}$ + 1.37 Å in our simulations, where $R_{surf}^{^{\prime}}$ is the distance between the centre of the metallic sphere and the nearest substrate atom of the dipole at $\vec{r}_{i}$. Here, we assume the same arrangement of the substrate atoms as mentioned in the previous section. The small factor of 1.37 Å is added to account for the fact that the effective image plane is slightly away from the outermost substrate atom. The value of 1.37 Å was taken from a previous study, wherein it was determined for a tungsten substrate^41^. The induced dipole moment is given by $\vec{\mu }_{im} = - \left( {\frac{{R_{surf}^{3} }}{{r_{i}^{3} }}} \right)\left[ {\vec{\mu }_{i} - \frac{{2\vec{r}_{i} \left( {\vec{r}_{i} \cdot \vec{\mu }_{i} } \right)}}{{r_{i}^{2} }}} \right]$, and the amount of the induced charge is given by $q_{im} = \left( {\frac{{R_{surf} }}{{r_{i}^{3} }}} \right)\vec{r}_{i} \cdot \vec{\mu }_{i}$. The interaction energy between the original dipole and the image dipole was calculated by using the abovementioned formula for $V_{dip - dip}$. The interaction energy between the original dipole and the image charge is calculated using the following formula^37^: $V_{charge - dip} = \left( {\frac{{q_{im} }}{{4\pi \epsilon_{0} r_{cd}^{3} }}} \right)\vec{r}_{cd} \cdot \vec{\mu }_{i}$. $\vec{r}_{cd}$ is defined as $\vec{r}_{cd} = \vec{p}_{im} - \vec{r}_{i}$, and $r_{cd} = \left| {\vec{r}_{cd} } \right|$. Using $V_{dip - dip}$ and $V_{charge - dip}$, we calculated the interaction energies between the original dipole and image charges/dipoles that include the images induced by other dipoles at the tip apex. It must be noted that the interaction between the image dipoles and dipoles at the molecules in the second layer is negligibly weak and does not affect the formation of the protrusion molecules. Hence, this fourth term representing the interaction energy is not crucial to our conclusion.

It should also be mentioned that the image-related dipole–dipole term is around two orders of magnitude stronger than the image-related charge–dipole term for our simulation conditions. Now, we compare our image-related dipole–dipole term with the formula presented in previous works^25,42,43^. A dipole on the surface and its image in the surface are necessarily on the same vertical line, so our image-related dipole–dipole formula appears to reduce to the following: $V_{dip - dip} = - \frac{{\mu^{2} }}{{16\pi \epsilon_{0} z^{3} }}$, where z [$= r_{ij} /2$] is the distance of the dipole centre from the image plane (which is assumed to be half the distance between the dipoles for consistency), and μ is the common dipole moment. However, the formula that appears in references^25,42,43^ is equivalent to $V_{dip - dip} = - \frac{{\mu^{2} }}{{32\pi \epsilon_{0} z^{3} }}.$ This implies that the predicted local bonding energy is smaller by a factor of around 2. Despite this discrepancy, we highlight that the forces calculated by both formulae are the same. For convenience, we call our formula V1 and the formula obtained in the previous studies V2. If we move a dipole with a small displacement of + *d* along an axis perpendicular to the surface, *r*_*ij*_ in the abovementioned $V_{dip - dip}$ formula becomes approximately 2*z* + 2*d*. Similarly, for a small displacement of – *d*, we obtain *r*_*ij*_ ≈ 2*z* − 2*d.* Hence, the differentiation of V_1_ can be approximately written as $\frac{{{\text{V}}1\left( {z + d} \right) - {\text{V}}1\left( {z - d} \right)}}{4d}$. In contrast, the differentiation of V2 is $\frac{{{\text{V}}2\left( {z + d} \right) - {\text{V}}2\left( {z - d} \right)}}{2d}$. Therefore, upon differentiation, our formula ends up providing the same results as those provided by the formula in references ^25,42,43^. As explained below, since we evaluated mean forces exerted on molecules in our simulations, both formulae should provide the same results.

For the fifth term, the total energy of a dipole is given by the following formula: $V_{dip} = - 0.5{\upalpha }F_{i}^{2}$^25,26^.

### Sixth term: the wind force

The wind force is given by the following formula: $\vec{F}_{wind} = \frac{{en_{e} \rho_{a} }}{{n_{a} }}\vec{J}$ , where *e* is the elementary charge, $n_{e}$ is the electron density, $n_{a}$ the density of impurity atoms, $\rho_{a}$ the impurity resistivity, and $\vec{J}$ the current density of field emission from a molecule. For $n_{e}$, we calculated the value for tungsten with its Fermi energy of 9.2 eV^44^, which is $1.27 \times 10^{23} {\text{/cm}}^{3}$. For $n_{a}$, we took the value for a solid C_60_, which is $1.44 \times 10^{21} {\text{/cm}}^{3}$^45^. For $\rho_{a}$, we took an experimentally obtained value from the reference^46^, which was $200\;\Omega \;{\text{cm}}$. To calculate the current density of the field emission, we employed the work function of 5.0 eV. This is because the previous work revealed that the work function of C_60_-covered metal surfaces was about this value for various metal surfaces^21^.

### Explanation of the algorithm of an optimization process

Using the six terms above, we calculated optimized molecule configurations. First, 500 molecules were randomly placed on the apex of a tip with its curvature at a radius of 160 nm. The area of molecules was limited within a polar angle of 3°, where the origin was situated at the centre of the sphere of the tip apex and the polar angle was measured from the tip axis. First, molecules were placed randomly one by one at a height of 5 Å from the tip surface. The distances of any two molecules were set to be more than 10 Å at the initial positions. If a molecule could not find a vacant space in the first layer, then it was placed in the second layer at a height of 15 Å from the surface. The same procedure was repeated until 500 molecules were placed. Thus we could make three layers. Then the molecule positions were relaxed and settled into optimized structures.

In the optimization process, we first, calculated the DC electric fields around the tip apex at each molecule position based on the method used in our previous work^20^. The electric fields induce dipole fields at molecules, which modulate the electric fields around the tip apex. In order to accurately calculate the modulated fields, one has to solve $3 \times N$ simultaneous equations^47^, where N is the number of molecules in our case. Here, in order to accelerate the simulations, we simply integrated the induced and the original fields at each molecule. The resulting fields were similar to those calculated by solving simultaneous equations within a difference of around 10%. We confirmed that there were no apparent differences in the resulting optimized structures between the two methods. The updated electric fields were used to calculate the dipole terms and the wind force.

By calculating all the six terms discussed above and using a conjugate gradient method based on the Polak-Ribiere algorithm^48,49^, the directions in which to move the molecules were determined. The lengths of the displacements were determined by a line minimization process^50^. All the molecules were displaced to new positions given by the calculated directions and lengths. At the new positions, the same procedures were applied to determine new positions, and the process was repeated until the mean forces exerted on molecules decreased below a certain threshold. The threshold was set around 100 times lower than the initial mean forces. Further reduction of the threshold did not dramatically change the resulting molecule configuration. Here, we defined the electric field *F*_*DC*_ as the value of the electric fields at the very top of the tip apex. The optimized structures were first simulated under *F*_*DC*_ of 0.5 V/nm. Then we simulated the optimized structures by gradually ramping up the *F*_*DC*_ such as 2 V/nm, 2.2 V/nm, 2.4 V/nm, … 3 V/nm, 3.1 V/nm, 3.2 V/nm … , 5 V/nm.

In the end, we would like to comment on two parameters whose values are scattering in some ranges among different pieces of literature. One is molecular polarizability. The molecular polarizability for a fullerene is scattering from 52.2 meV/(V/nm)^2^ (i.e. ${\upalpha }/4{\uppi }\epsilon_{0}$ = 75.1 Å^3^) to 106.3 meV/(V/nm)^2^ (153.1Å^3^) in different works^38,51^. Here, we employed 106.3 meV/(V/nm)^2^ (153.1Å^3^), but we also tested with molecular polarizability of 60.1 meV/(V/nm)^2^ (86.5Å^3^)^52^. With this lower value, molecules do not change their positions, and the formations of protrusions on a weakly-bound layer, such as the ones discussed in Fig. 6b, were not observed below 3 V/nm. In contrast, we observed some spots in the emission patterns below 3 V/nm in our experiments, as shown in Figs. 2 and 3, which indicate formations of some protrusions on the weakly-bound layer. Since the lower molecular polarizability did not reproduce our observations, we employed the highest value. Additionally, the impurity resistivity $\rho_{a}$ of $3.8\;\Omega \;{\text{cm}}$ was calculated for a fullerene molecule in different literature^36^, which is far lower than the value of $200\;\Omega \;{\text{cm}}$ in our simulations. We have also tested this value. In this case, the weakly-bound molecules evaporate at around 3.7 V/nm. This change did not affect our conclusion.

## References

[1] Sessoli R, Gatteschi D, Caneschi A, Novak MA (1993). Magnetic bistability in a metal-ion cluster. Nature.

[2] Gatteschi D, Sessoli R, Villain J (2006). Molecular Nanomagnets.

[3] Westerström R (2012). An endohedral single-molecule magnet with long relaxation times: DySc2N@C80. J. Am. Chem. Soc..

[4] Huang T (2011). A molecular switch based on current-driven rotation of an encapsulated cluster within a fullerene cage. Nano Lett..

[5] Peller D (2020). Sub-cycle atomic-scale forces coherently control a single-molecule switch. Nature.

[6] Gross L (2011). Recent advances in submolecular resolution with scanning probe microscopy. Nat. Chem..

[7] Gomer R (1993). Field Emission and Field Ionization.

[8] Rose DJ (1956). On the magnification and resolution of the field emission electron microscope. J. App. Phys..

[9] Müller EW (1950). Die Sichtbarmachung einzelner Atome und Moleküle im Feldelektronenmikroskop. Z. Naturforschung A.

[10] Becker JA, Brandes RG (1955). On the adsorption of oxygen on tungsten as revealed in the field emission electron microscope. J. Chem. Phys..

[11] Melmed AJ, Müller EW (1958). Study of molecular patterns in the field emission microscope. J. Chem. Phys..

[12] Neo Y, Matsumoto T, Tominita M, Sasaki M, Mimura H (2012). Necessary conditions for two-lobe patterns in field emission microscopy. Jpn. J. Appl. Phys..

[13] Matsumoto T, Nakamura T, Neo Y, Mimura H, Tomita M (2011). Graphene Simulation.

[14] Rezeq M, Joachim C, Lwin MH, Navarro FA, Grill L, Joachim C (2013). Observations of individual Cu-phthalocyanine molecules deposited on nano-tips in the field emission microscope. Imaging and Manipulating Molecular Orbitals Advances in Atom and Single Molecule Machines.

[15] Esat T, Friedrich N, Tautz FS, Temirov R (2018). A standing molecule as a single-electron field emitter. Nature.

[16] Girifalco LA (1992). Molecular properties of C60 in the gas and solid phases. J. Phys. Chem..

[17] Drechsler M, Müller EW (1952). Die Bestimmung der Polarisierbarkeit von Atomen und Molekülen mit dem Feldelektronenmikroskop. Z. Phys..

[18] Murphy EL, Good RH (1956). Thermionic emission, field emission, and the transition region. Phys. Rev..

[19] Young RD (1959). Theoretical total-energy distribution of field-emitted electrons. Phys. Rev..

[20] Yanagisawa H (2010). Laser-induced field emission from a tungsten tip: Optical control of emission sites and the emission process. Phys. Rev. B.

[21] Tsuei K-D (1997). Photoemission and photoabsorption study of C60 adsorption on Cu(111) surfaces. Phys. Rev. B.

[22] Li W (2014). Fullerene film on metal surface: Diffusion of metal atoms and interface model. Appl. Phys. Lett..

[23] Gall’ NR, Rut’kov EV, Tontegode AY (2004). Interaction of C60 molecules with the (100)W surface: Adsorption, initial stages of film growth, and thermal transformation of the adsorption layer. Semiconductors.

[24] Bonetto F, Vidal RA, Quintero Riascos V, Bonin CJ, Ferrón J (2017). Growth, thermal desorption and low dose ion bombardment damage of C60 films deposited on Cu(111). J. Phys. Commun..

[25] Maschhoff BL, Cowin JP (1994). Corrected electrostatic model for dipoles adsorbed on a metal surface. J. Chem. Phys..

[26] Tsong TT, Kellogg G (1975). Direct observation of the directional walk of single adatoms and the adatom polarizability. Phys. Rev. B.

[27] Forbes RG, Orloff J (2008). Gas field ionisation sources. Handbook of Charged Particle Optics.

[28] Mayer A (2007). Formulation in terms of normalised propagators of a charge-dipole model enabling the calculation of the polarisation properties of fullerenes and carbon nanotubes. Phys. Rev. B.

[29] Fiks VB (1959). On the mechanism of the mobility of ions in metals. Sov. Phys. Solid State.

[30] Huntington HB, Grone AR (1961). Current-induced marker motion in gold wires. J. Phys. Chem. Solids.

[31] Araidai M, Watanabe K (2007). Ab initio calculation of surface atom evaporation in electron field emission. e-J. Surf. Sci. Nanotechnol..

[32] Gruznev D (2013). Stepwise self-assembly of C_60_ mediated by atomic scale moiré magnifiers. Nat. Commun..

[33] Sato M (1980). Gas adsorption on tungsten exposed to a mixture of nitrogen and oxygen. Phys. Rev. Lett..

[34] Giannozzi P (2009). QUANTUM ESPRESSO: A modular and open-source software project for quantum simulations of materials. J. Phys. Condens. Matter.

[35] Giannozzi P (2017). Advanced capabilities for materials modelling with quantum ESPRESSO. J. Phys. Condens. Matter.

[36] van der Hamada I (2014). Waals density functional made accurate. Phys. Rev. B.

[37] Jackson JD (1999). Classical Electrodynamics.

[38] Shuai Z, Brédas JL (1993). Erratum: Electronic structure and nonlinear optical properties of the fullerenes C60 and C70—A valence-effective-Hamiltonian study. Phys. Rev. B.

[39] Forbes RG (1977). Atomic polarisability values in the SI system. Surf. Sci..

[40] Santos FC (2004). The electrostatic field of a point charge and an electrical dipole in the presence of a conducting sphere. Eur. J. Phys..

[41] Hall BM, Tong SY, Mills DL (1983). Large-angle electron-energy-loss spectroscopy with the inclusion of a surface image potential. Phys. Rev. Lett..

[42] Forbes RG (1989). On different types of dipole–dipole interaction. J. Phys..

[43] Forbes RG (1989). On charged surface models and the origins of field adsorption. Surf. Sci..

[44] Islam MF, Malik FB (2009). Enhancement of laser-induced field emission in tungsten due to a metastable d band. Solid State Commun..

[45] Martinek J, Stankowski J (1996). A model of fullerene conductance. Solid State Commun..

[46] Makarova TL (2001). Electrical properties of two-dimensional fullerene matrices. Carbon.

[47] Gravil PA (1995). Polarization of C60 by the surface electric field of GeS(001). Surf. Sci..

[48] Fletcher R, Reeves CM (1964). Function minimization by conjugate gradients. Comput. J..

[49] Polak E, Ribière G (1969). Note sur la convergence de méthodes de directions conjuguées. Rev. Fr. Informat Rech. Opér..

[50] Press WH, Teukolsky SA, Vetterling WT, Flannery BP (2007). Numerical Recipes: The Art of Scientific Computing.

[51] Jonsson D, Norman P, Ruud K, Ågren H, Helgaker T (1998). Electric and magnetic properties of fullerenes. J. Chem. Phys..

[52] Norman P, Luo Y, Jonsson D, Ågren H (1997). Ab initio calculations of the polarizability and the hyperpolarizability of C60. J. Chem. Phys..

